# Exoskeleton use in post-stroke gait rehabilitation: a qualitative study of the perspectives of persons post-stroke and physiotherapists

**DOI:** 10.1186/s12984-020-00750-x

**Published:** 2020-09-10

**Authors:** Julie Vaughan-Graham, Dina Brooks, Lowell Rose, Goldie Nejat, Jose Pons, Kara Patterson

**Affiliations:** 1 Department of Physical Therapy, University of Toronto, 160-500 University Avenue, Toronto, ON M5G 1V7 Canada; 2 School of Rehabilitation Science, McMaster University, 1400 Main St. W, Hamilton, ON L8S 1C7 Canada; 3 Department of Mechanical Engineering, University of Toronto, 5 King’s College Road, Toronto, ON M5S 3G8 Canada; 4 Department of Mechanical and Industrial Engineering, Robots for Society, Autonomous Systems and Biomechatronics Laboratory, University of Toronto, Toronto, Canada; 5 Legs & Walking Lab, Shirley Ryan Ability Lab, Department of Physical Medicine & Rehabilitation, Feinberg School of Medicine, Department of Biomedical Engineering & Department. of Mechanical Engineering, McCormick School of Engineering, Northwestern University, 355 E. Erie St, Chicago, IL 60611 USA

**Keywords:** Exoskeletons, Stroke, Biomedical engineering, Rehabilitation

## Abstract

**Background:**

Wearable powered exoskeletons are a new and emerging technology developed to provide sensory-guided motorized lower limb assistance enabling intensive task specific locomotor training utilizing typical lower limb movement patterns for persons with gait impairments. To ensure that devices meet end-user needs it is important to understand and incorporate end-users perspectives, however research in this area is extremely limited in the post-stroke population. The purpose of this study was to explore in-depth, end-users perspectives, persons with stroke and physiotherapists, following a single-use session with a H2 exoskeleton.

**Methods:**

We used a qualitative interpretive description approach utilizing semi-structured face to face interviews, with persons post-stroke and physiotherapists, following a 1.5 h session with a H2 exoskeleton.

**Results:**

Five persons post-stroke and 6 physiotherapists volunteered to participate in the study. Both participant groups provided insightful comments on their experience with the exoskeleton. Four themes were developed from the persons with stroke participant data: (1) Adopting technology; (2) Device concerns; (3) Developing walking ability; and, (4) Integrating exoskeleton use. Five themes were developed from the physiotherapist participant data: (1) Developer-user collaboration; (2) Device specific concerns; (3) Device programming; (4) Patient characteristics requiring consideration; and, (5) Indications for use.

**Conclusions:**

This study provides an interpretive understanding of end-users perspectives, persons with stroke and neurological physiotherapists, following a single-use experience with a H2 exoskeleton. The findings from both stakeholder groups overlap such that four over-arching concepts were identified including: (i) Stakeholder participation; (ii) Augmentation vs. autonomous robot; (iii) Exoskeleton usability; and (iv) Device specific concerns. The end users provided valuable perspectives on the use and design of the H2 exoskeleton, identifying needs specific to post-stroke gait rehabilitation, the need for a robust evidence base, whilst also highlighting that there is significant interest in this technology throughout the continuum of stroke rehabilitation.

## Introduction

Over the period 1990–2017 there has been a 3% increase in age-standardized rates of global stroke prevalence [1] and a 33% decrease in mortality due to improved risk factor control and treatments [2]. Therefore, stroke survivors are living longer with mild to severe lifelong disabilities requiring long term assistance [1]. As a result, stroke presents a significant socioeconomic burden accounting for the largest proportion of total disability adjusted life years (47.3%) of neurological disorders [3]. Walking impairments, one aspect of stroke disabilities, negatively impact independence and quality of life [4], and recovery of walking is a primary goal post-stroke [5].

Wearable powered exoskeletons are a new and emerging technology originally developed as robots to enable persons who were completely paralyzed due to spinal cord injury to stand and walk [6, 7], but more recently developed to provide sensory-guided motorized lower limb assistance to persons with gait impairments [8]. They require the active participation of the user from the perspective of integrating postural control/balance and the locomotion pattern in real life environments whilst simultaneously providing assistance to achieve typical lower limb movement patterns in a task specific manner [8]. The Exo-H2 is a novel powered exoskeleton in that it has six actuated joints, the hip, knee and ankle bilaterally, and uses an assistive gait control algorithm to provide lower limb assistance when the gait pattern deviates from a prescribed pattern [9]. As stroke impairments typically influence hip, knee and ankle movements the H2 was considered an appropriate exoskeleton for our study [8, 10].

Significant limitations persist in current exoskeleton designs such as weight, cost, size, speed and efficiency [11]. Although end-users’ perspectives are essential in the design and development of assistive technology [12, 13], there is a paucity of literature from both persons with disabilities and physiotherapists (PTs) perspectives [14, 15]. Over the last decade end-user perspectives have primarily been studied in spinal cord injury (SCI) in which four studies used semi-structured interviews [16–19], and 3 studies used survey methods [20–22] with sample size ranging from 3 to 20 persons. However, these studies included both complete and incomplete SCI with most participants being non-ambulatory representing a very different end-user population compared to persons post-stroke. A further two studies reported end-user perspectives using survey methods with persons with multiple sclerosis (MS) [23], and persons with MS, SCI or acquired brain injury (ABI) [24]. Wolff et al.,(2014) utilized an online survey to evaluate perspectives on potential use of exoskeletons with wheelchair users, primarily persons with SCI, and healthcare professionals, but no PTs were included [25]. To date only one study by Read et al.,(2020) specifically investigated perspectives of 3 PTs on exoskeleton use using semi-structured interviews with persons with SCI or stroke. Currently, a mixed-methods study is underway to investigate perspectives of PTs and persons with stroke [26]. Thus, further research is needed to explore in-depth, utilizing a qualitative research approach, end-users’ perspectives on lower limb exoskeleton use in post-stroke gait rehabilitation.

It is important to understand and incorporate end-user perspectives [27], persons post-stroke and physiotherapists, with respect to the design of exoskeletons and their implementation to effectively facilitate uptake both in clinical practice and community settings. Therefore, the purpose of our study is to explore the perspectives of persons post-stroke and physiotherapists following a 1.5 h single-use session with a H2 exoskeleton.

## Methods

We used a qualitative interpretive description approach utilizing semi-structured in-person interviews, with persons post-stroke and neurological physiotherapists, following a 1.5 h session with a H2 exoskeleton. The interpretive description approach aims to develop clinically relevant knowledge within the practice context by advancing the understanding of experiential knowledge [28](p.23–51) [29]. Interpretive description draws upon an inductive reasoning approach generating empirical disciplinary knowledge that is credible, meaningful and clinically relevant [30].

### Study sample and recruitment

The study sample consisted of two participant groups: (i) persons with stroke (PWS); and, (ii) their physiotherapist/s (PT). A purposive sampling strategy was used to select individuals participating in post-stroke gait rehabilitation within a private community out-patient rehabilitation setting.

Participants were recruited from three private community based neurological rehabilitation locations in the greater Toronto area. The inclusion/exclusion criteria are outlined in Table 1. On recruitment of the PWS, with their permission, their treating PT was contacted and invited to observe their client’s H2 exoskeleton session and participate in a separate semi-structured interview. Our study was reviewed and approved by the University of Toronto Health Services Ethics Committee.

**Table 1:** Inclusion / Exclusion Criteria

Inclusion Criteria	Exclusion Criteria
Adults aged between 30 and 75 years	Previous history of neurologic disease such as Parkinson’s disease, pontine and/or cerebellar lesions, peripheral neuropathies
Adult-onset of a 1st cerebrovascular accident (CVA) with resulting hemiparesis	Botox injection to the lower limb musculature within the last 12 weeks
> 12 weeks < 2 years post neurological injury	Receptive Aphasia
Able to maintain standing for at least 60 s on the less affected lower limb, and within their stability limits, perform a reaching activity with the less affected upper limb	Orthopedic lower extremity pathology or rheumatoid conditions which affects the ability to sit, stand or walk including hip, knee, ankle contractures
Able to walk for 6 min with or without assistance of 1 person	Auditory or visual deficits that could prevent data collection
Capacity to independently consent	Cardiovascular conditions incompatible with intensive gait training
Able to understand instructions in English	

### Data collection

Prior to the H2 exoskeleton session, each PWS attended an assessment session during which descriptive and evaluation measures were recorded. Descriptive measures included the National Institutes of Health Stroke Scale (NIHSS) [31] and Chedoke-McMaster Stroke Assessment (CMSA) – Leg and Foot scales [32]. The NIHSS comprises a 13-item scale with a maximum score of 42. The higher the score the greater the level of neurological deficit. The CMSA leg and foot scale each comprise of levels 1–7, the lower the level the greater the degree of neurological deficit. Evaluation measures included the 10 m walk test (10MWT) [33], 6 min walk test (6minWT) [34], and the Brief Balance Evaluation Systems Test (Brief-BESTest) [35]. These descriptive and evaluation measures were used to characterize the PWS participant group (refer to Table 2). None of the PWS had prior experience with an exoskeleton.

**Table 2:** Persons with Stroke Demographics, Descriptive & Clinical Measures

Demographics							Descriptive Measures			Clinical Measures		
ID #	Sex M/F	Age (yrs)	Diagnosis	Time since diagnosis (months)	Brace	Gait Aid	NIHSS Composite Score	CMSA Leg	CMSA Foot	10MWT Self selected (m/s)	6minWT (Metres)	Brief-BESTest Composite Score
1	M	54	R CVA	10	L AFO	R Trekking pole	5	4	2	0.65	188	6
2	M	75	L CVA	6	N/A	L Trekking pole	5	6	5	0.4	135	6
3	M	38	R CVA	7.5	N/A	4 point cane	1	6	5	0.62	161	11
4	M	65	R CVA	19	L AFO	R trekking pole	4	3	3	0.82	236	10
5	M	69	L CVA	15	N/A	N/A	1	6	6	1.43	370	18

Key

*M* Male, *F* Female, *R/L CVA* Right/Left Cerebrovascular accident, *R/L AFO* Right/Left Ankle Foot Orthosis, *R* Right, *L* Left, *N/A* Not Applicable, *NIHSS* National Institutes of Health Stroke Scale, *CMSA* Chedoke McMaster Stroke Assessment, *10MWT* 10 m walk test (self-selected velocity), *6minWT* 6 min walk test, *Brief-BESTest* Brief Balance Evaluation Systems Test

Furthermore, each PT participant completed a ‘Professional Profile Form’ providing demographic information including education, duration of clinical experience in neurological rehabilitation, duration at current work location, previous experience with exoskeletons, and use of technology in clinical practice (refer to Table 3).

**Table 3:** Physiotherapist participant demographics

PT ID #	Sex M/F	Education	Clinical experience NeuroRehab (years)	Current work setting (years)	Practice Setting	Previous Exoskeleton experience	Technology use in practice Yes/No
PT-01	F	MSc	4	1	Private Community	1 year	Yes FES & BWST
PT-02	F	MSc	10	8	Private Community	1 day Exo trial	Yes FES & BWST
PT-03	M	MSc	3	3	Private Community	1 day Exo trial	Yes FES & BWST
PT-04	M	MSc	2	2	Private Community	1 day Exo trial	Yes FES & Exo
PT-05	F	PhD	3	3	Private Community	N	Yes FES & Balance assessments
PT-06	F	MSc	1.5	0.5	Private Community	Y 9 months with an exo	Yes FES

Key

*PT* Physiotherapist, *M* Male, *F* Female, *NeuroRehab* Neuro-rehabilitation, *Exo* Exoskeleton, *MSc* Master of Science, *PhD* Doctor of Philosophy, *FES* Functional Electrical Stimulation, *BWST* Body-weight Support Treadmill

The H2 exoskeleton session consisted of each PWS attending a single session in a university laboratory with their PT. Our PT researcher answered any questions that the participants had and provided guidance to the PWS to familiarize them with the exoskeleton. The H2 exoskeleton has a pre-programmed gait pattern with two stepping modes: a single step mode and a continuous gait mode. Both modes, and stop, were controlled via a controller input from a smartphone application by a member of the research team (LR). The don/doffing of the H2 exoskeleton was required to be completed in standing. Once the PWS had been fitted into the exoskeleton they were familiarized with the single step mode initially, and once comfortable, progressed to the continuous gait mode. Each PWS spent approximately 45–60 min walking in the H2 exoskeleton. Speed and assistance while walking in the H2 exoskeleton were adjusted depending upon the PWS ability, however manual support via both upper limbs was necessary by the primary researcher and the PWS PT for the entire duration of all PWS exoskeleton sessions. PWS and PTs attended interviews individually, organized at their convenience approximately 5–10 days following their experience with the exoskeleton. An interview guide, (refer to Additional files 1 and 2), were utilized for both participant groups to prompt discussion however, the interview was semi-structured allowing the participant to elaborate on their specific thoughts and ideas. Each interview was audio-recorded and transcribed verbatim by an independent transcriptionist.

### Data analysis

The data from the two participant groups, PWS and PT, were analyzed separately. The interview transcripts were imported into the NVivo qualitative software program to facilitate the coding and analytic process. Preliminary coding was undertaken by the first author (JVG). Coding was a progressive iterative process, beginning initially with labeling segments of the data retaining as close as possible to the participant’s wording, termed invivo codes. As data analysis progressed with successive transcripts, data was reassembled into categories through making comparisons between participants as well as comparing data from the same participant. To ensure the trustworthiness of the data analysis, two members of the research team (DB)(KP) reviewed the codebook such that categories and themes were developed by consensus between the three research team members. In addition, this process was documented through the writing of memos and the chronological development of a code book such that all codes, categories and themes were illustrated in the original data.

Invivo codes from the PWS participant group such as ‘exo for therapy’, ‘novel treatment tool’ and ‘psychological benefits’, were grouped together and recoded as ‘Adjunct to Rehab’, which in turn was grouped under the category ‘Therapeutic Device’. Further analysis and interpretation of this data led to the development of the theme ‘Integrating exoskeleton use’. Invivo codes from the PTs included, ‘Easy to access and use’, ‘Ability for PTs to adjust parameters’ and ‘Gait parameter settings per patient’, these codes were grouped together forming the category ‘Device interface’, which in turn was grouped under the theme ‘Device programming’. Tables 4 & 5 illustrate the codes, categories and thematic development for both the PWS and PT participant groups.

**Table 4:** Persons with Stroke Thematic Development

In-vivo Code	Category	Theme
Expectations	Client engagement	Adopting Technology
Machine-body disconnect	Client Dissonance	
Augmenting my function		
Exo appearance	Appearance	Device Concerns
Exo improvements		
Exo comfort	Comfort	
Exo control options	Control Options	
Fitting concerns	Fitting Issues	
Exo purchase	Cost & Availability	
Walk program	Not a natural walk	Developing walking ability
Movement interference		
Increasing walking activity	Potential benefits	
Limiting compensations		
Adjunct to rehab	Therapeutic device	Integrating exoskeleton use
Early stage rehab		
Learning to use the device		
Daily use	Everyday device	
Community use

**Table 5:** Physiotherapist Thematic Development

In-vivo Code	Category	Theme
New technology	Technology in development	Developer - User Collaboration
Expectations		
Differing knowledge	Knowledge base	
Exo evidence base		
Patient program	Training	
Physio program		
Battery	Device Hardware	Device Concerns
Pelvic attachment		
Foot plates		
Motors		
Weight of the device		
Alignment	Impact on alignment & movement	
Movement		
Price	Cost	
Manpower needs		
Assistive device	Augmentative vs. Robot	Device Programming
Exo gait pattern		
Robot-exo programs		
Machine Learning		
One person use	Individualized vs. multi-user	
Multi-person use		
Fitting settings		
Easy to access and use	Device Interface	
PT to adjust parameters		
Gait parameter settings per patient		
Exo data reports	Data Output	
Exo data to guide PT		
Balance difficulties	Physical	Clinical characteristics requiring consideration
Coping with muscle tone		
Standing Tolerance		
Able to don independently		
Cognitive deficits	Cognitive, Perceptual, Communication	
Communication and comprehension		
Safe to use	Safety & Interest	
Patient interest		
Risk re injuring patient		
When should the exo be used	Care Continuum	Indications for use
Patient criteria		
Outside of therapy		
Integration into clinical practice	Feasibility of use in clinical practice	
Impact on therapy time		
Time to fit device		
Hands on		
Positive aspects of the Exo	Therapeutic Tool	
As an exercise training tool		
Improve exercise tolerance		
To assist in everyday activities		

## Results

Five persons with stroke and 6 physiotherapists volunteered to participate in the study.

### Participants with stroke (refer to Table 4)

Four themes were developed from the PWS participant data: (1) Adopting technology; (2) Device concerns; (3) Developing walking ability; and (4) Integrating exoskeleton use.

#### Adopting technology

The PWS described their willingness to try the new technology but, at the same time, their disappointments. Two categories were developed: a) client engagement; and, b) client dissonance which incorporated 3 codes: i) expectations; ii) machine-body disconnect; and, iii) augmenting my function. One PWS describes:

“I like technology, I’m kind of an early adopter of various technologies, so I would definitely be a proponent”. (PWS participant #02)

However, there was some disappointment with the exoskeleton especially with respect to expectations as one PWS stated:

“Well, to be honest with you I was a little disappointed, it’s not what I expected, it didn’t feel like a proper walking pattern”. (PWS participant #01)

Whilst others described there being a disconnect between the exoskeleton and their body as one PWS describes:

“It’s exaggerating a lot of the movement, like a normal movement wouldn’t do that, I feel like it’s something like if I walked on the moon, it feels that way” (PWS participant #03).

The PWS participants described wanting the exoskeleton to assist and correct their movement rather than walking for them:

“I just thought it wasn’t helping me, it was just doing the walking for me. If there was a device that was an assist, that would be very useful training outside of therapy” (PWS participant #01)

The data identified that the PWS were excited to try this new technology and recognized its potential use in stroke rehabilitation. However, they did not like the pre-programmed walking pattern, stating that they would prefer for their gait to be assisted, but they could appreciate the value of having a device that could assist their walking training outside of therapy.

#### Device concerns

Each of the PWS provided insightful comments and suggestions related to multiple aspects of the exoskeleton and these were grouped under the following categories: a) Appearance; b) Comfort; c) Control options; d) Fitting issues; and, e) Cost and availability. One of the PWS commented:

“We don’t want to look like a robot, we want to be as normal as possible, it’s too bulky, it’s not something that I can wear day to day … I want to be able to wear it under clothing”. (PWS participant #03)

Whilst another appreciated the issues around appearance but conceded:

“It wouldn’t be your preference *(the appearance)*, it wouldn’t be your favorite thing, but you would do it if it gave you mobility”. (PWS participant #02)

Control options revolved around wanting control on the actual device, not on a separate device such as smart phone as dropping the phone, or losing the controller, would be a significant issue particularly if the PWS was using the device alone. Fitting issues such as needing help to don the device, the time taken and the weight of the device were significant concerns for all PWS participants. It took approximately 30–40 min to fit the device to each PWS, causing one PWS to comment:

“It was laborious, it would have to become less cumbersome, less lengthy process if it was going to be used effectively.” (PWS participant #04)

Whilst another put it in perspective of a regular treatment session:

“I am paying for one hour of physiotherapy, so if it take 30 minutes just to put it on then I would rather focus on doing the actual therapy rather than trying to fit it”(PWS participant #03)

Lastly, all PWS discussed cost and availability of the device as a significant factor that will affect uptake by end users and rehabilitation facilities and suggestions were made for alternate funding options rather than purchase. One PWS suggested:

“Perhaps renting it for a defined period of time so that you could use it to train your gait pattern and maybe train your stamina and your speed.” (PWS participant #02)

The PWS had specific suggestions with respect to exoskeleton design and control, as well as identifying usability issues such as time and assistance required to don the device and cost.

#### Developing walking ability

Two paradoxical categories were developed under the theme ‘Developing walking ability’: a) Not a natural walk; whilst also identifying the potential of these devices in stroke rehabilitation, b) Potential benefits.

The PWS commented that the exoskeleton walk did not feel like a natural walking pattern and that in some instances the exoskeleton seemed to interfere with their ability to walk.

One of the PWS stated:

“It felt unnatural, it would lift your leg, it kind of threw the foot back a little and then forward … like it’s pulling your leg back before it pushes it forward.” (PWS participant #02)

And another reported:

“It interfered with the weight transfer between one leg and the other, I found it much harder to get on either leg when I was taking a step because I couldn’t load properly” (PWS participant #05)

Although the PWS identified these movement problems they could also anticipate potential benefits of these devices such as increasing walking distance and activity levels as one describes:

“I think I would be able to walk longer and further with it” (PWS participant #01).

Another benefit of improving the rhythm and pattern of their gait was highlighted as one participant stated:

“You will be able to move with a more rhythmical gait pattern, not so compensatory, so you won’t have to unlearn bad habits.” (PWS participant #04).

So, while the PWS acknowledged the potential of exoskeleton use to improve their walking pattern, they highlighted that the preprogrammed walking pattern of the exoskeleton was not optimal.

#### Integrating exoskeleton use

This theme comprises two categories illustrating the PWS different perspectives on using the device as: a) Therapeutic device; and, b) Everyday device. The category ‘Therapeutic device’ comprises three codes: i) Learning to use the device; ii) Early stage rehab; and, iii) Adjunct to rehab. The participants discussed everyday use from the perspective of: i) Daily use; and, ii) Community use.

One of the PWS describes the difficulty he experienced when he first put the exoskeleton on and the impact it had on his walking,

“In the beginning I found it very difficult, very difficult, but the longer I was on it and the more I walked the easier it came to me, but still it felt not quite natural, it interfered with me trying to improve my current gait pattern.” (PWS participant #04).

The PWS discussed the potential of exoskeletons in both the early and later stages of stroke rehabilitation and that they felt these devices would be a useful adjunct to rehabilitation, as one notes:

“I would have liked to have tried it back then … when I couldn’t walk … it would have been more meaningful to me.” (PWS participant #05).

Whilst another stated:

“It is something (an exoskeleton) that should be pursued in terms of availability in rehabilitation and outside rehabilitation.” (PWS participant #02).

In addition, to the exoskeleton being a therapeutic device, the PWS also reported that they would like an exoskeleton that they could use on a daily basis and potentially in the community. However, they identified a number of concerns and limitations that would have to be overcome in order for this to become a reality. One of the participant’s highlighted his concerns about the ability to use an exoskeleton in inclement weather:

“I don’t know how water resistant this thing is and different temperatures too, so what if the device goes and malfunctions because it’s -30°C outside, what am I to do? So I would have reservations about going out in public.” (PWS participant #03).

Another participant highlighted the functional limitations of the current device, stating:

“I would be looking for something that has much greater functional ability, if it can walk forward and then upstairs without having to switch it around, change something, to be able to get on a streetcar … if it assists automatically then it may be a real benefit”. (PWS participant #05).

The PWS identified their desire for an exoskeleton with a wide range of applications from being a therapeutic device applicable across the continuum of care to a device that could assist their functional ability within the community.

In summary, the PWS were excited to try this new technology, all recognized the potential of these devices for stroke rehabilitation, however, to facilitate uptake by PWS essential improvements in key areas were identified.

### Physiotherapist participants (refer to Table 5)

Five themes were developed from the PT participant data: (1) Developer-User collaboration; (2) Device specific concerns; (3) Device programming; (4) Patient characteristics requiring consideration; and, (5) Indications for use.

#### Developer-user collaboration

The PT participants discussed the need for ‘Developer-User Collaboration’ from three perspectives: a) Technology in development; b) Knowledge base; and, c) Training. The PTs acknowledged the potential of exoskeleton technology for post-stroke gait rehabilitation but they felt that this technology is currently in its infancy, as one PT commented:

“I think right now with Exos we’re at the same stage that IBM was when they were using a room to have one computer …. so I think we need to get to the MacBook Pro version of one … I think we will get there eventually but we’re just not there yet.” (PT participant #04).

Whilst another PT described what she thinks it will take for this technology to actually gain traction in the clinical community:

“To be able to adjust and modify and apply *(the exoskeleton)*, like a slip on slip off kind of thing, I can see benefits of that, I see it future, future, if we had a tool that could facilitate that I think that would be great, but in its current state there is a lot of work still to be done.” (PT participant #05).

The PTs highlighted the gap between the clinical knowledge required to know what is needed of exoskeletons in order to be able to integrate them into clinical practice, and the engineering technical knowledge required to build these devices, and that a greater degree of interaction between these two domains of knowledge is required, as one PT noted;

“There is kind of like a gap, people are developing these devices, and people are trying to use these devices, but the crossover between them maybe is part of the problem.” (PT participant #01).

The PTs also discussed the need to develop a clinically relevant evidence base with respect to the exoskeleton, as one commented:

“I would want to understand how effective of a treatment option is this compared to what else is currently available. Like, how much more feasible, affordable is it compared to what we as therapists already do, perhaps what we have is even more easily accessible, more affordable etc.” (PT participant #04).

The need for training, ongoing support and perhaps a designated person in their facility that has a specialist knowledge base of the exoskeleton were all highlighted as necessary components of integrating these devices into clinical practice. One PT suggested:

“I would say a week-end course to start, but then you would need ongoing support personnel to reach out to …. ongoing training and development to really get buy-in.” (PT participant #05).

The PTs also discussed that there would need to be a training program for the PWS as well, that they would need to learn how to use the device, as one commented:

“I would say probably 10 sessions, probably because the first few sessions you are probably playing with all the settings so you are constantly changing the device on the patient and it’s hard for them to get used to it” (PT participant #02).

The PTs identified from their perspective that exoskeleton technology is still in its infancy in terms of being able to meet the needs of clinical practice as well as the evidence-base, that there is a need for greater collaboration between developers and end-users, and training and device specific support will be required for clinical integration.

#### Device specific concerns

A number of ‘Device specific concerns’ were identified by the PTs which were grouped into the following three categories: a) Device Hardware; b) Impact on alignment and movement; and, c) Cost.

With respect to the exoskeleton hardware the PTs identified the battery pack, pelvic attachment, foot-plates, motors and the weight of the device all requiring consideration for future development. One PT suggested;

“You would want some kind of gauge that tells them at this speed there is this much battery left.” (PT participant #01)

Whilst another was very dissatisfied with the pelvic attachment, and commented:

“If I can be blunt, I thought it *(the pelvic attachment)*, was useless, the actual attachment of the (hip) motor to the pelvic component where is it applying the torque, I feel it would need to be revised in some way.” (PT participant #05).

The PTs did not like the foot-plate attachments due to their size, lack of adjustability between patients, and the rigidity of the foot-plate. One of the PTs made the following suggestion:

“Could there be a special kind of shoe that fits the exoskeleton, the patient would just have to buy their own that fits in and out with a pressure sensitive sole, which would provide us with valuable data. We need a flexible foot-plate to gain a typical gait pattern so it could allow the foot to move like it would in walking.” (PT participant #01).

The PTs liked having motors at the hip, knee and ankle but would like to be able to adjust how much each motor is augmenting the patient’s movement, as one PT commented:

“I think it is good to have all 3 motors, but it would be good to kind of control what it’s doing at each joint rather than all of it on at the same time, the same force for both limbs.” (PT participant #06).

There were concerns about how the exoskeleton influenced the patient’s gait pattern due to the alignment of the hip/pelvis and knee and subsequent impact on the base of support, as well as the weight of the battery shifting the patient’s centre of mass backwards. One of the PTs explained:

“It doesn’t follow our typical alignment of our anatomy, the angle of our femurs to the pelvis and this influences the gait pattern in particular the weight transfer between the legs.” (PT participant #01).

Whilst another commented:

“My patient had difficulty going to full extension at the hip, and that just threw off the pattern and then he was doing all these other compensatory movements that he wouldn’t normally do.” (PT participant #02).

Lastly, the PTs were concerned about the purchase cost of these devices, as well as the cost of implementation, how many facilities and/or how many patients would actually be able to afford them and so would the device be feasible for a very small percentage of the stroke population, as one PT commented:

“We are thinking about our *(facility)* budgets, and what would we have access to within our current budgets, and so if we’re lucky we might be able to have one for across our entire programs, but then how difficult is it going to be to access, book and use, because anything that takes a lot of time to organize to use is going to be an automatic barrier.” (PT participant #05).

Another PT remarked:

“Like a handful maybe, maybe able to afford this, and for them weighing the pros and cons of how much this is really facilitating their quality of gait versus without, and is that benefit worth what it is going to cost.” (PT participant #06).

In summary, the PTs liked that there were motors for the three lower limb joints, provided suggestions on device improvements, but voiced concerns about the cost of these devices which from their perspective would influence uptake in clinical practice.

#### Device programming

The theme ‘Device Programming’ was developed as the PTs identified specific clinical needs of exoskeleton technology. This theme comprises four categories: a) Augmentative vs. Robot; b) Individualized vs. multi-user; c) Device interface; and, d) Data output.

All of the PTs discussed the need for an augmentative device, not a robot. They clearly expressed that they do not want to use a device with their clients that replaces their gait pattern with a pre-programmed gait pattern, they stated that they want to be able to fine tune assistance on a limb by limb, joint by joint basis to optimize their client’s gait pattern. One PT explained,

“Things that they could have recovered such as swing or stance, we were telling them to let the machine do it, so we were taking away their active movements and were making it more of a cognitive gait pattern, so we were kind of taking them away from what we want them to get.” (PT participant #01).

Another PT commented,

“There was this battle almost between the patient and the device that the patient knew how they walk and then the device is trying to impose a walk on them.” (PT participant #03).

The PTs wanted a device that they could tailor to the needs of their specific client, as one noted,

“It’s really important to have the type of technology that actually adapts to the person’s need versus trying to make every patient fit the same shoe.” (PT participant #05).

Differing needs of the device were discussed dependent upon whether the device was going to be used for one person, or whether the device was to be used in a rehabilitation facility setting. Ease of set up between patients, and the ability to retrieve patient specific settings in terms of the actual hardware and software were identified as critical to using exoskeletons in clinical practice, as one PT commented,

“If we are using one device in a clinic we would have to change it between each person, that would eat up therapy time.” (PT participant #01).

Another PT suggested,

“Would the exo be something that could be plugged into an iPad and then you just pull up the patient identifier … the parameters are set as you figured it out in the initial assessment and you just select …” (PT participant #06)

The PTs agreed that they wanted to be able to make adjustments to their client’s gait pattern on an as needed basis, and therefore the device interface needs to be user-friendly, accessible, and preferably available on a smart phone, iPad or tablet via an App as one PT noted,

“They would walk a lap and then we would see what we want to change about it and we should be able to adjust as much as we would like to.” (PT participant #03).

The PTs also commented that they do not want to just be able to adjust joint angles, that they want to be able to adjust the temporal aspects of movement as well such as swing and stance time, as one describes,

“Could you make it so that you didn’t just adjust the settings of like the joint angles and the length of the femur … but also, like some people have a longer swing pattern, if you could adjust those types of things as you went as well?” (PT participant #01).

The PTs also discussed the data output that they would like to have from the exoskeleton, and that they wanted this data not only to measure outcomes but to also guide therapy, as one PT explained,

“If the foot-plates sense pressure, so where on their feet were they landing, or putting their weight? Because sometimes it’s obvious and other times it’s not.” (PT participant #03).

Whilst another described how data output could be used to demonstrate improvements,

“If you can adjust the parameters at each joint, then we should be able to track that right, so if they start off with 70% assistance at the hip, but then they go down to 40% that would show improvement.” (PT participant #02).

In summary, the PTs identified specific desires related to the nature of the assistance provided by the exoskeleton, how they could adjust or interface with the gait pattern and how that might differ if the device was for single vs. multi-use as well as discussing some of the data they would like from the device in order to be able to track outcomes and progress.

#### Clinical characteristics requiring consideration

The PTs discussed aspects of the clinical presentation that would require consideration and agreed that determining what clinical characteristics are important for successful use of the exoskeleton is essential to its uptake and usefulness in clinical practice. The theme ‘Clinical characteristics requiring consideration’ comprises three categories: a) Physical; b) Cognitive / Perceptual / Communication; and c) Safety and interest.

One of the PTs discussed the issue of balance in the device,

“Their balance was worse in it than out of it and so putting somebody …. in trialing this on somebody who has less balance than the people that we did, I don’t feel like I need to see that, I think I know where that would go.” (PT participant #01).

Also, the issue of atypical muscle tone was raised, and concerns about how the exoskeleton would respond, as one PT commented,

“It didn’t respond well to (*atypical*) tone, I think that is a real challenge, if your hip flexors start contracting very quickly, like in a patient with hyper-reflexia, and the device thinks you’re sitting, but the patient isn’t sitting, or somebody starts ‘clonusing’ what would happen to the device?” (PT participant #01).

The PTs highlighted that clients would have to have a significant amount of standing tolerance, even to just don the device due to the time involved, as well as the limitation of many patients only having one functional upper limb. One PT questioned,

“What is the basic level of mobility that a patient needs to have in order to have something like this trialed on them and for it to be effective?”

Whilst another commented,

“A lot of them don’t have a functional arm for even getting into or out of it especially if you have to stand to do it.” (PT participant #03).

Cognitive, perceptual and communication deficits were also raised as probable barriers to using an exoskeleton. The PTs acknowledged that there is a lot of interest by patients in exoskeletons and the PTs believe exoskeletons will be a positive adjunct to rehabilitation both in and out of the clinical environment. However, there were safety concerns as patients required more assistance to walk in the exoskeleton due to its impact on their balance, as well as the potential for musculoskeletal injuries, as one PT explained,

“I think it has a long way to go before I feel comfortable putting something like this on somebody who may not even have the core strength to sit up yet, and kind of throwing them through the motions, I think it would be possibly placing them at risk of injury.” (PT participant #05).

To summarize, the PTs highlighted the need to determine the critical aspects of the clinical presentation for successful device use, and to ensure patient safety.

#### Indications for use

The PTs discussed a wide range of applications for exoskeleton technology which developed into the theme ‘Indications for use’ and comprises three categories: a) Care continuum; b) Feasibility of use in clinical practice; and, c) Therapeutic tool.

The PTs could foresee a future role for exoskeleton use throughout the continuum of care as well as outside of formal rehabilitation. One PT commented,

“I think if money wasn’t an option … having it available right through (the continuum of care) would be nice because definitely not everyone would be good to use this in the acute stage, plus the people who fatigue quickly, or those we want to stop the development of compensatory patterns, I think the exo has the potential to help all those neuro patients.” (PT participant #01).

Time was the most discussed factor in terms of using an exoskeleton in clinical practice, the time for training, the time required to book the device and any assistants for a specific therapy session, the time to fit the device, and the time to make the required adjustments would all impact on the PTs decision as to whether the device is used in clinical practice, as one explained,

“Like realistically … like I will devote 10-15 minutes of my session to set it up for that patient, if what I’m getting out of it (treatment effectiveness) is worthwhile.” (PT participant #03).

The PTs agreed that exoskeletons will be a therapeutic tool at some stage in rehabilitation for some patients. They discussed limiting the development of compensatory patterns when walking, augmenting motor function of the more affected hip, knee and ankle for persons with stroke, and an opportunity to train a more typical gait pattern, as one PT described,

“What these devices give us is intensity of practice right …. but also practice in a gait pattern that is a more typical gait pattern rather than a compensatory gait pattern.” (PT participant #05).

However, the PTs felt that this technology is not ready yet for clinical practice, as one noted:

“I love the idea, the futuristic component, but it felt like the computers of the 80’s, it kind of gave you that reminiscent feel of like this is a clunky, big device, and then how smooth and portable and sexy almost they (computers) are now and I can see the potential into the future, so that’s exciting.” (PT participant #06).

In summary, the PTs could envisage wide application of exoskeleton technology given the potential for increased intensity of optimal practice, but highlighted that time demands are a significant limitation and that further development of the technology is required.

## Discussion

This study explored the perspectives of persons with stroke and their physiotherapists using in-depth semi-structured interviews following a single-use experience with a H2 exoskeleton. To date only one study using qualitative research methods has specifically investigated physiotherapist perspectives of exoskeleton use in persons with stroke [14]. Currently, no literature using qualitative research methods exists on the perspectives of persons with stroke with respect to exoskeleton technology. Whilst the PTs and PWS data were analyzed separately, the themes developed from each group overlap such that four over-arching concepts will be discussed relative to the findings including: (i) Stakeholder participation; (ii) Augmentative vs. Autonomous Robot; (iii) Exoskeleton usability; and (iv) Device specific concerns.

### Stakeholder participation

Although there is a large body of evidence supporting the inclusion of end-user perspectives both in retail marketing and design of medical devices to ensure that products and devices meet end-user needs [12], end-user perspectives with respect to assistive technology, such as the exoskeleton for post-stroke gait rehabilitation, appears to be lacking [14, 36]. The literature suggests that it is important that end-users have an actual exoskeleton experience as perceptions of, and experience with exoskeleton technology are potentially quite different [20]. End-users should include persons with disabilities as well as rehabilitation clinicians as stakeholder groups to ensure the breadth of perspectives. For example, in our study the PTs discussed the need for Developer-User Collaboration, whilst the PWS discussed their willingness as well as their disappointment in the technology, both stakeholder groups were vocal on their device concerns, the need for an augmentative device rather than an autonomous robot, and their views on how exoskeletons could be integrated into clinical practice and daily life.

Our findings suggest the PWS and PTs can provide insightful knowledge with respect to exoskeleton use and design to assist in its future development. Knowledge translation in neurological rehabilitation is complex with considerable challenges with respect to developing, sustaining and implementing multi-professional perspectives [37–39], as well as a surprising lack of inclusion of persons with disabilities in the knowledge translation process. Additionally, there are inherent challenges in finding suitable professional and industry partners, as well as challenges in developing, funding and publishing mixed method research studies that explore not only the quantitative effects of a device, such as reported in a recent review on quantitative performance evaluation of these technologies [40], but also explore the perspectives of end-users. Thus, the development of strategic engineering - end user partnerships is essential to optimize the uptake of exoskeleton technology.

### Augmentation vs. autonomous robot

Exoskeleton technology was originally developed for individuals whom had no walking capacity [41], for example, a person with a complete spinal cord injury and thus total loss of motor function below the level of their lesion. However, due to the increasing prevalence of incomplete spinal cord injuries [42] and application of exoskeleton technology in stroke rehabilitation [8], augmentation of the individual’s walking pattern is desired, rather than replacement with a programmed walking pattern. Our findings suggest that PWS are excited and hopeful about exoskeleton technology, however, were disappointed that the technology did not yet meet their expectations. They talked about a disconnect between their body and the machine, that the machine imposed a walk on them, but that they did not want to be walked, they wanted a device that augmented their function. Likewise, the PTs in this study echoed the sentiments of the PWS in that they hoped for an augmentative and adaptive device, not an autonomous robot. As the majority of persons post-stroke maintain some motor control of their lower limbs it is essential that PTs are be able to easily adjust the amount of assistance provided at any one joint, in real-time specific to the individual, to optimize that person’s gait pattern. Our study participants highlighted that the exoskeleton programmed walking pattern did not feel like, or look like, a natural walk, which over time could result in inappropriate neuromuscular plastic adaptation [43, 44]. So, although the end users acknowledged the potential benefits of exoskeleton use, specifically, increasing walking activity both within and outside of formal rehabilitation whilst simultaneously limiting compensatory motor behavior, benefits that are supported by the neuromuscular plasticity and motor learning evidence base [43, 45], are dependent on the exoskeleton being able to augment or reproduce a typical gait pattern specific to the individual.

### Exoskeleton usability

The study participants highlighted a number of significant determinants of uptake within a clinical facility including: availability of the device and manpower resources; cost and maintenance requirements; ease and time to fit the device; user-friendly device interface; ability to store an individual’s fitting measurements and gait parameters for ongoing use; data output to demonstrate change in motor behavior; access to resource personnel; and, ease to switch between multiple users. Whereas, for a single use device appearance, sound, ability to don/doff independently, battery life, device interface, cost, maintenance, and ability to use within a home and community setting were paramount. Whilst the current literature identifies that user interface is critical to clinical and patient uptake [12], our study findings broaden our understanding of the many aspects of this technology that require consideration to optimize uptake.

Locomotion is often viewed as a lower body activity, with little to no consideration of the postural control and balance requirements for successful locomotion, rather than a whole body activity [46, 47]. The inclusion criteria for gait rehabilitation technologies, for example body-weight support treadmill (BWST) training, tend to be limited to lower limb motor function scores, walking speed and level of dependence when walking [48]. Interestingly, the PTs in this study expressed reservations about PWS not having sufficient postural control to use an exoskeleton for gait rehabilitation, and the potential risk of injury or development of compensatory motor behavior. Significant reservations were also identified on exoskeleton use for PWS with cognitive, perceptual and/or communication deficits. Therefore, consideration of what percentage of persons with neurological deficits would benefit from exoskeleton technology is essential from a return on investment perspective.

The study findings highlighted the need for a robust evidence base to support exoskeleton use. Therefore, it is essential to define: (i) Client selection criteria for whom exoskeleton technology would be most beneficial; and, (ii) Intent of the device, for example to facilitate the walking pattern in a person who is not yet walking, to improve the walking pattern in a person who is already walking, and/or to improve walking tolerance / community accessibility. Unless client selection criteria and goals of device use are clearly established prior to seeking to establish effectiveness, there is the potential risk of exoskeleton technology suffering a similar fate to body-weight support treadmill trials’ lack of statistically significant effect [49–51]. Likewise, although some attempts have been made for persons with spinal cord injuries [52], the development of optimal exoskeleton training protocols are required as there is currently no evidence on how long it takes a person to become comfortable using an exoskeleton.

Lastly, our stakeholder groups identified potential use of exoskeletons both in the early and later stages of rehabilitation. However, the PWS in some instances had differing opinions on exoskeleton use, as one participant explained that he would use a device to help him walk no matter how it felt or what it looked like, whilst another stated that how it looked, sounded and felt to him was very important and that he would not currently wear an exoskeleton in a public place. This highlights the individual nature of a person’s perspective and the importance that the range of voices are heard for exoskeletons to appeal to all users [36, 53].

### Device specific concerns

Comfort, cost, battery life, ease of putting on/taking off the device, energy cost and functional use were device specific concerns identified by both stakeholder groups and are consistent with the literature [25]. Hip-foot alignment which is critical to efficient weight transfer during walking [54], was highlighted as a specific concern by the PTs, as they noted that the H2 exoskeleton does not follow the anthropometric dimensions of the human skeleton. This potentially increases the users dependence on a gait aid or physical assistance, which was particularly noticeable in our study as our PWS were independent ambulators, some requiring the use of a gait aid and/or brace, but all required the assistance of two persons via their upper limbs to walk safely during their exoskeleton session.

Both stakeholder groups concurred with the literature that an exoskeleton with six actuated joints was optimal for individuals with neurological-movement related problems [55]. However, the role of the ankle/foot in balance and locomotion has not garnered a significant amount of attention such that actuation of the ankle joint remains an inconsistent feature [55]. The neurophysiology literature suggests an important role of the sole of the foot in postural control, balance and stance to swing transitions [56–60]. The PTs in our study were concerned that this essential aspect in the recovery of balance, postural control and locomotion was not been adequately addressed in exoskeleton design and suggested that the inclusion of a flexible, pressure-sensitive footplate would greatly enhance clinical uptake.

A limitation of this research is the small number of participants in both stakeholder groups. Additionally, there was a short time delay between some end users H2 experience and their interview. This may have resulted in their recall not being a true reflection of their experience. However, the participants use of the H2 exoskeleton was a novel experience and the researcher conducting the interviews was present for each users experience and took field notes to support the interview. Lastly, the perspectives of the participants is specific to their single-use experience with the H2 exoskeleton. Further research is warranted with larger sample sizes and differing types of exoskeletons.

## Conclusions

This study simultaneously explored the perspectives of five PWS and their PTs following a single-use experience with the H2 exoskeleton. The findings of this study demonstrate that both stakeholder groups have valuable contributions to the ongoing development of exoskeleton technology and therefore the inclusion of end user perspectives is essential to optimize uptake. Our study findings have highlighted the unique needs of the stroke population with respect to user selection criteria, the need for an augmentative device that reflects current anatomical and neurophysiological knowledge as well as a robust evidence base, whilst also identifying there is an appetite for this technology throughout the continuum of care in stroke rehabilitation. Further study is warranted on PWS and PTs perspectives on exoskeleton technology utilizing larger samples and different exoskeletons.

## Supplementary information

**Additional file 1.** Stroke Participant Interview Guide & Instructions.

**Additional file 2.** Physiotherapist Interview Guide & Instructions.

## Data Availability

The data sets used and/or analyzed during the current study are available from the corresponding author on reasonable request.

## Abbreviations

ATD-PA: Assistive Technology Device Predisposition Assessment
PWS: Persons with stroke
PT/PTs: Physiotherapist/s
CVA: Cerebrovascular accident
NIHSS: National Institutes of Health Stroke Scale
CMSA: Chedoke-McMaster Stroke Assessment
10MWT: 10 m walk test
6minWT: 6 min walk test
Brief-BESTest: Brief Balance Evaluation Systems Test
