# Supplementary material for: Exoskeleton use in post-stroke gait rehabilitation: a qualitative study of the perspectives of persons post-stroke and physiotherapists
Source: J Neuroeng Rehabil. 2020 Sep 10;17:123. doi: 10.1186/s12984-020-00750-x (PMC7488039; doi:10.1186/s12984-020-00750-x)
Supplement: Supplementary file 1 — Additional file 1. Stroke Participant Interview Guide & Instructions. [file 12984_2020_750_MOESM1_ESM.docx]

**Additional file 1: Stroke Participant Interview Guide & Instructions**

Interview Instructions

Thank you for taking the time to talk with me today.

I am interested in talking with you about your recent experience using the exoskeleton. I would like to know what your thoughts are about this device from putting it on and taking it off, how it looks and feels, your thoughts on whether you would wear it in the community, to how it assisted your walking, as well as your thoughts about whether this kind of device would be a helpful tool during the rehabilitation process.

I do not have any set expectations as to how you should feel about this device, how it looks or its usefulness. I am interested in your honest opinion.

I will be audio-recording this interview.

Please let me know if this is clear or if you have any questions.

## Interview guide

Open ended questions will be used as these have a greater tendency to yield rich data, for example:

“Tell me about….”, “How…..”, “What……”, “When…..”, “Could you describe further”

“Could you tell me about how the device felt…..”

Some examples of potential questions:

1. How did you find the fitting process of the exoskeleton?
2. Is it something that you think you would consider putting on, on a daily basis?
3. How it did feel once you had the exoskeleton on?
4. Was the exoskeleton comfortable? If not, what made it uncomfortable?
5. How easy or difficult was it to walk in the exoskeleton?
6. What did you like about the exoskeleton?
7. What didn’t you like about the exoskeleton?
8. Would you consider wearing this device in the community? If not, why not?
9. Do you think the exoskeleton would have been a useful device during your in-patient rehabilitation? If so, why? Or why not?
10. Do you think the exoskeleton would be a useful device during your out-patient rehabilitation? If so, why? Or why not?
11. Would you consider purchasing a device such as an exoskeleton for your continued use?
12. What changes, if any, do you think would improve the usability of this device?
