# Supplementary material for: Exoskeleton use in post-stroke gait rehabilitation: a qualitative study of the perspectives of persons post-stroke and physiotherapists
Source: J Neuroeng Rehabil. 2020 Sep 10;17:123. doi: 10.1186/s12984-020-00750-x (PMC7488039; doi:10.1186/s12984-020-00750-x)
Supplement: Supplementary file 2 — Additional file 2. Physiotherapist Interview Guide & Instructions. [file 12984_2020_750_MOESM2_ESM.docx]

**Additional file 2: Physiotherapist Interview Guide & Instructions**

Interview Instructions

Thank you for taking the time to talk with me today.

I am interested in talking with you about your thoughts on the use of exoskeletons in stroke rehabilitation and their use within the community post rehabilitation. I would like to know what your thoughts are about the feasibility of using an exoskeleton during both in-patient and out-patient rehabilitation. I’m interested to know what your thoughts are about the time and ease to put on and take off the device, how the device is fitted as well as the learning that will be required by the PT to use such a device safely in rehabilitation. I’m interested to know what your thoughts are with respect to the facilitators and barriers to use exoskeletons throughout the rehabilitation process and what kind of information would you like to be able to get from these devices to assist you in making decisions about how best to improve your client’s walking performance.

I do not have any set expectations as to how you should feel about this device, its usefulness or not. I am interested in your honest opinion.

I will be audio-recording this interview.

Please let me know if this is clear or if you have any questions.

## Interview guide

Open ended questions will be used as these have a greater tendency to yield rich data, for example:

“Tell me about….”, “How…..”, “What……”, “When…..”, “Could you describe further”

“Could you tell me …..”

Some examples of potential questions include:

1. What was your opinion of the fitting process of the exoskeleton?
2. How likely would it be for you to learn about this new technology and integrate it into your clinical practice?
3. What do you think would make it difficult to use in clinical practice?
4. What would make it easier in order for it to be used in clinical practice?
5. During which phase of the rehabilitation process do you think an exoskeleton would be most useful? Acute inpatient, post-acute inpatient, out-patient, community?
6. Do you think the exoskeleton influenced the gait pattern of your client?
7. If this device was available for you to use with your patients during their rehabilitation do you think you would use it?
8. What did you like about the exoskeleton?
9. What didn’t you like about the exoskeleton?
10. What changes, if any, do you think would improve the usability of this device?
11. What information would be useful to you as a physiotherapist to be able to get from the exoskeleton?
